# Ablation of Dicer from Murine Schwann Cells Increases Their Proliferation while Blocking Myelination

**DOI:** 10.1371/journal.pone.0012450

**Published:** 2010-08-27

**Authors:** Juliane Bremer, Tracy O'Connor, Cinzia Tiberi, Hubert Rehrauer, Joachim Weis, Adriano Aguzzi

**Affiliations:** 1 Institute of Neuropathology, University Hospital of Zurich, Zurich, Switzerland; 2 Functional Genomics Center Zurich, University of Zurich, Zurich, Switzerland; 3 Institute of Neuropathology, Medical Faculty, RWTH University Aachen, Aachen, Germany; Universidade Federal do Rio de Janeiro, Brazil

## Abstract

The myelin sheaths that surround the thick axons of the peripheral nervous system are produced by the highly specialized Schwann cells. Differentiation of Schwann cells and myelination occur in discrete steps. Each of these requires coordinated expression of specific proteins in a precise sequence, yet the regulatory mechanisms controlling protein expression during these events are incompletely understood. Here we report that Schwann cell-specific ablation of the enzyme Dicer1, which is required for the production of small non-coding regulatory microRNAs, fully arrests Schwann cell differentiation, resulting in early postnatal lethality. Dicer^−/−^ Schwann cells had lost their ability to myelinate, yet were still capable of sorting axons. Both cell death and, paradoxically, proliferation of immature Schwann cells was markedly enhanced, suggesting that their terminal differentiation is triggered by growth-arresting regulatory microRNAs. Using microRNA microarrays, we identified 16 microRNAs that are upregulated upon myelination and whose expression is controlled by Dicer in Schwann cells. This set of microRNAs appears to drive Schwann cell differentiation and myelination of peripheral nerves, thereby fulfilling a crucial function for survival of the organism.

## Introduction

Proper myelination is essential for the efficient saltatory conduction of action potentials, the trophic support of axons, and the maintenance of axonal integrity in the peripheral nervous system (PNS). Defective PNS myelination occurs in hereditary peripheral neuropathies [1]. Sporadic peripheral neuropathies can arise due to a wide variety of factors, including metabolic disorders (e.g. diabetes mellitus), intoxication (e.g. alcohol), and autoimmune disorders (e.g. Guillain-Barré syndrome) [2]. Treatment of peripheral neuropathies is still unsatisfactory in most cases, and is likely to benefit from increased knowledge about peripheral myelin development and maintenance.

The mature myelin sheaths wrapped around the large-diameter axons of peripheral neurons arise from neural crest cells which subsequently develop into Schwann cell precursors (SCPs), immature Schwann cells, and finally mature myelinating or non-myelinating Schwann cells. Each stage of Schwann cell development is associated with a set of specific protein markers, the expression of which is thought to be driven primarily by axon-derived signals at the SCP stage and by the secretion of autocrine survival factors at the Schwann cell stage (reviewed in [3]). This precise developmental program requires tightly regulated transcriptional and post-transcriptional control of protein expression, the details of which are still incompletely understood.

One post-transcriptional mechanism that appears to be critical for the proper development of numerous tissues is the microRNA (miRNA) system [4]. miRNAs are short (20–30 nucleotides) non-coding RNAs which are processed from endogenously expressed pri-miRNAs by the enzyme Drosha. The resulting stem-loop pre-miRNAs are exported to the cytoplasm where they are further processed by the enzyme Dicer [5], unwound into single-stranded miRNAs, and loaded into the RNA-induced silencing complex (RISC). The miRNA-loaded RISC then binds to complementary miRNA recognition sequences in the 3′untranslated regions (UTRs) of specific target mRNAs. The primary function of the miRNA system appears to consist of mRNA silencing. Target mRNAs are either degraded, or their translation is inhibited. A single miRNA can have multiple mRNA targets, which allows for broad miRNA-mediated regulation of expression programs (reviewed in [6]). Genetic ablation of Dicer in mice is embryonic lethal, illustrating the indispensable role that miRNAs play during development [7]. Furthermore, studies utilizing tissue-specific expression systems have revealed a vital role for miRNAs in the development of specific organ systems (reviewed in [8]). In the nervous system, miRNAs appear to be important for the development of Purkinje [9] and forebrain [10] neurons, oligodendrocyte differentiation and central nervous system (CNS) myelination [11], [12], [13], and as shown more recently, also for peripheral myelination by Schwann cells [14].

In order to determine which miRNAs might be required for peripheral myelination, we created a mouse line undergoing Schwann cell-specific deletion of Dicer1, by crossing mutants whose endogenous Dicer1 sequences were flanked by *floxP* sites (Dicer1^fl/fl^) to mice expressing the Cre recombinase under the control of the desert hedgehog promoter (Dhh-Cre).

## Results

### Dicer depletion in Schwann cells leads to arrest at the pro-myelin stage and impairs myelination

**Figure 1:**
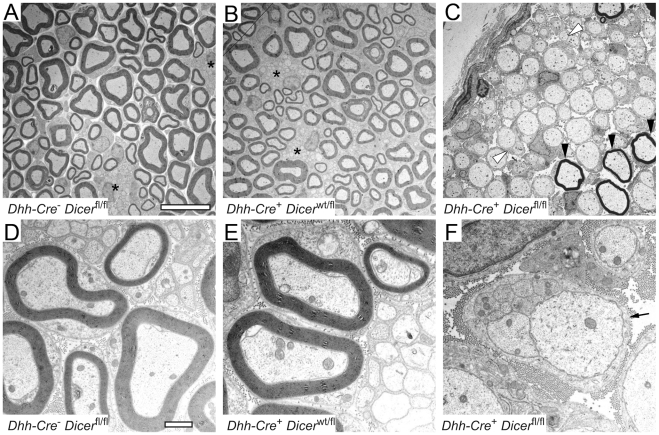
Defective myelination following Schwann cell specific Dicer depletion. Electron microscopy of sciatic nerves derived from 18-day old Dicer^fl/fl^ Dhh-Cre^+^ (**C and F**) and control Dicer^wt/fl^ Dhh-Cre^+^ (**B and E**) and Dicer^fl/fl^ Dhh-Cre^−^ (**A and D**) littermates. Low magnification figures (**A–C**) show normally myelinated nerves fibers in control mice (**A and B**) and normal Remak bundles of unmyelinated small-caliber axons (black stars). In Dicer depleted mice (**C**), most of the large caliber axons remain unmyelinated; only a few fibers are ensheathed by a thin myelin sheath (black arrowheads). Most of these nerve fibers show proper axonal sorting by Schwann cells; only a few large-caliber axons remain unsorted (white arrowheads). Higher magnification figures show normal myelinated and unmyelinated nerve fibers (**D and E**) in controls. A Schwann cell in Dicer^fl/fl^ Dhh-Cre^+^ mice containing non-sorted and unmyelinated large caliber axons is shown. Basal lamina formation by this Schwann cell is evident (black arrow), indicating development into immature Schwann cell (**F**). Scale bar in A–C = 10 µm and in D–F = 1 µm.

In order to achieve Schwann cell-specific deletion of the enzyme Dicer, we bred Dicer^fl/fl^ mice to mice expressing Cre-recombinase specifically in Schwann cells (tg*Dhh-Cre*, henceforth termed Dhh-Cre^+^). The Cre recombinase in these mice is already active in Schwann cells of the precursor stage at embryonic day 12/13 (E12/13) [15]. In contrast to their littermates, Dicer^fl/fl^ Dhh-Cre^+^ mice lacking Dicer expression in Schwann cells exhibited a severe behavioral phenotype characterized by ataxia and hind limb paresis. In compliance with animal welfare regulations, mice were euthanized at the age of 25 days. Electron microscopy (EM) of 18-day old Dicer^fl/fl^ Dhh-Cre^+^ sciatic nerves revealed a severe myelination defect when compared to control littermates Dicer^wt/fl^ Dhh-Cre^+^ and Dicer^fl/fl^ Dhh-Cre^−^. In Dicer^fl/fl^ Dhh-Cre^+^ sciatic nerves, most fibers remained unmyelinated; the few myelin sheaths present were abnormally thin (**Fig. 1**). Most Dicer-depleted Schwann cells properly sorted axons, resulting in the typical 1∶1 Schwann cell to axon ratio. However, some bundles of Dicer mutant nerves containing axons >1µm, which would normally be sorted and myelinated, remained unsorted (**Fig. 1c and 1f**).

**Figure 2:**
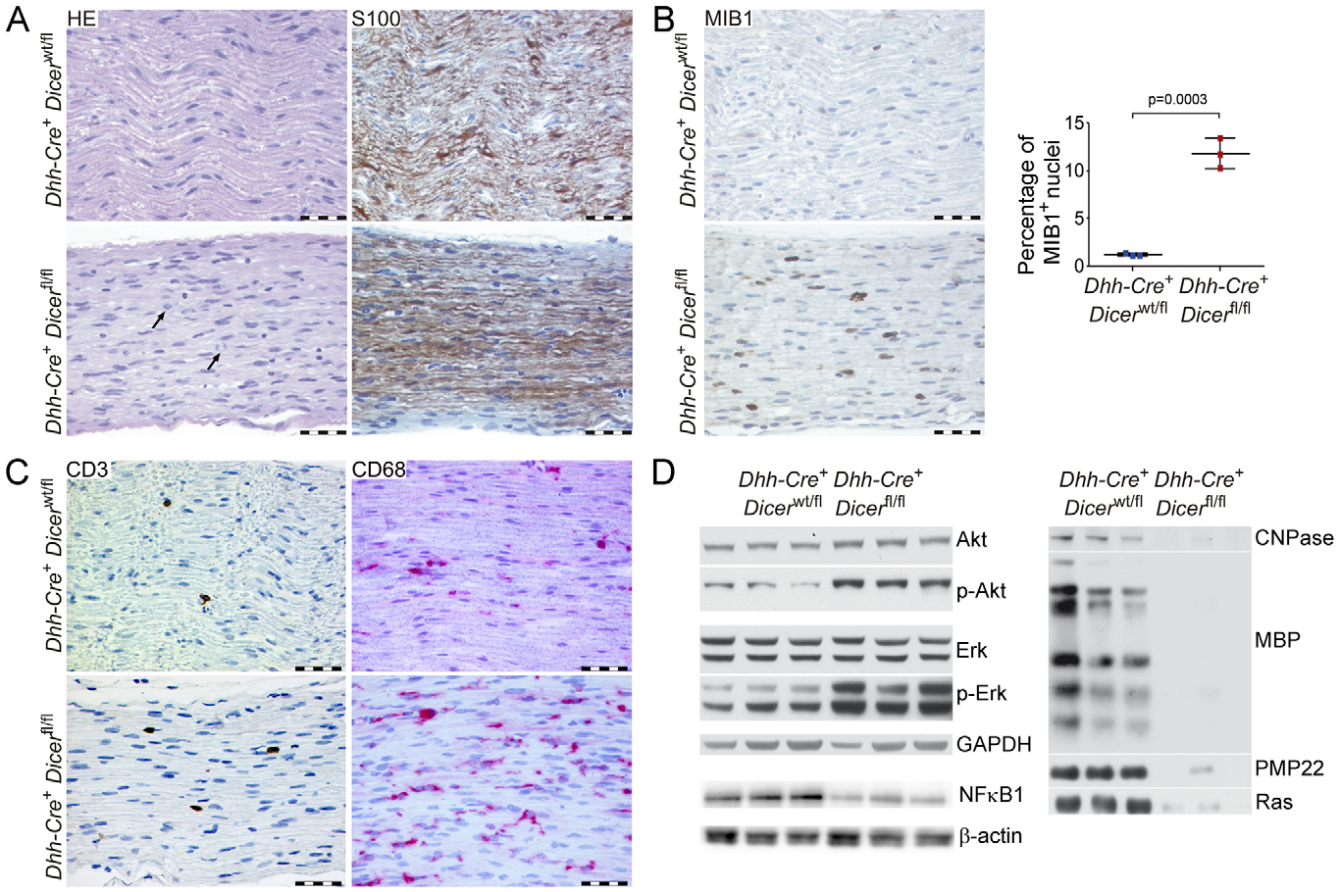
Histological and biochemical analysis of mice lacking Dicer in Schwann cells. (**A–C**) Longitudinal sections of sciatic nerves from 22-day old Dicer^fl/fl^ Dhh-Cre^+^ and control Dicer^wt/fl^ Dhh-Cre^+^ littermates stained with haematoxylin and eosin (HE) or decorated with S100, MIB1, CD3, or CD68 antibody. At least four nerves were analyzed per genotype. Scale bars = 50 µm. S100 positivity indicates Schwann cell development progresses at least up to the stage of immature Schwann cells (**A**). Increased proliferation in Dicer mutant nerves, as indicated by mitotic events (black arrows in **A**) and increased percentage of positive nuclei in MIB1 immunohistochemistry (**B**). Quantification of MIB1-positive nuclei shows a significantly higher percentage of proliferating cells in Dicer^fl/fl^ Dhh-Cre^+^ compared to Dicer^wt/fl^ Dhh-Cre^+^ littermate nerves. Error bars indicate standard deviation, *p* = 0.0003, p value was determined using unpaired two-tailed student's t-test (**B**). Few CD3-positive T cells and an increased percentage of CD68-positive macrophages infiltrated the nerves of Dicer^fl/fl^ Dhh-Cre^+^ mice (**C**). Biochemical analysis of signal transduction pathways and myelin components by Western blot (**D**). Compared to control Dicer^wt/fl^ Dhh-Cre^+^ littermates, phospho-Akt and phospho-Erk were significantly increased in sciatic nerves of 18-day old mice lacking Dicer in Schwann cells, while total Akt and Erk protein levels were unchanged compared to controls, and NFκB was significantly decreased. In agreement with the histological findings, components of non-compact (CNPase) and compact myelin (MBP, PMP22) were nearly absent from Dicer mutant nerves. In addition, Ras levels were significantly lower in Dicer mutant nerves. GAPDH and β-actin served as loading controls.

We did not observe normal Remak bundle formation in Dicer mutant nerves. Small-caliber axons remained in groups that also contained large caliber axons. In contrast to normal Remak bundle formation [16], groups of small-caliber axons and were engulfed by Schwann cells as a whole, and axons were not individually ensheathed and separated from each other by Schwann cell processes (mesaxons). In addition, the number of these immature axon bundles was far lower than the number of Remak bundles in control nerves, indicating that unmyelinated nerve fiber development was also severely disturbed in these nerves. To determine at which stage myelin development was blocked in Dicer^fl/fl^ Dhh-Cre^+^ mice, we compared myelin markers between Dicer^fl/fl^ Dhh-Cre^+^ and Dicer^wt/fl^ Dhh-Cre^+^ nerves with immunohistochemical and biochemical techniques (**Fig. 2**). Components of mature non-compact myelin like 2′,3′- cyclic nucleotide 3′-phosphodiesterase (CNPase), and of compact myelin, like myelin basic protein (MBP) and peripheral myelin protein 22 (PMP22), were nearly undetectable in Dicer^fl/fl^ Dhh-Cre^+^ nerves via immunoblot (**Fig. 2d**).

Schwann cells were not absent in Dicer^fl/fl^ Dhh-Cre^+^ nerves, as evidenced by EM and by positive S100 immunoreactivity in nerves of 22-day old mice (**Fig. 2a**). Furthermore, EM analysis of Schwann cells in Dicer mutant nerves showed basal lamina formation by Schwann cells (**Fig. 1f**). These findings indicate that Schwann cells in Dicer mutant nerves developed at least until the stage of immature Schwann cells. Proper sorting of the majority of axons suggested an arrest at the pro-myelin stage. Development of myelinating Schwann cells is known to be accompanied by cessation of Schwann cell proliferation. In contrast to Dicer^wt/fl^ Dhh-Cre^+^ nerves, Dicer mutant nerves showed evidence of mitotic events and a significantly increased percentage of Schwann cells expressing proliferation marker MIB1 (Ki67; 11.8±0.9% in Dicer mutant Schwann cells versus 1.22±0.07% in controls, *p* = 0.0003; **Fig. 2b**). Based on immunohistochemistry, we determined that sciatic nerves of Dicer mutant mice contained no B-cells (B220) and only rare T-cells (CD3; 1.6±0.6% positive cells per total number of nuclei in Dicer^fl/fl^ Dhh-Cre^+^ nerves versus 1.6±0.2% in controls, *p* = 0.96, **Fig. 2c**). An increased prevalence of macrophages was detected in Dicer mutant nerves (CD68; 11.3±3.5% positive cells per total number of nuclei in Dicer^fl/fl^ Dhh-Cre^+^ nerves versus 7.2±2.4% in controls, *p* = 0.04, **Fig. 2c**). Although we observed an increased number of macrophages in Dicer mutant nerves, the percentage was in a similar range in Dicer mutant and controls. Based on this and based on their morphology with elongated nuclei, we conclude that the proliferating cells were indeed Schwann cells. In parallel to increased proliferation, Dicer mutant nerves at p22 showed increased cell death as determined by TUNEL staining (<0.05% TUNEL positive nuclei in all control nerves and ca. 2% in Dicer mutant nerves). Erk and Akt signal transduction pathways which are known to regulate myelination were significantly altered in 18-day old mutant nerves. Both Akt and Erk phosphorylation were significantly increased. Furthermore, Ras and NFκB protein expression was significantly lower in Dicer mutant nerves compared to controls (**Fig. 2d**).

**Figure 3:**
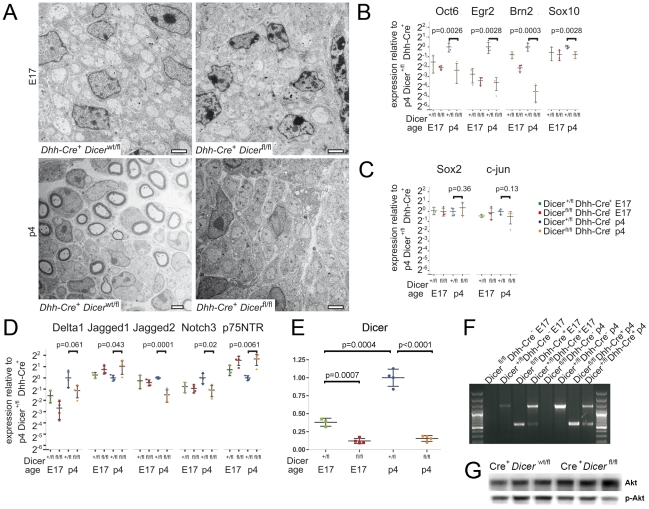
Time course analysis of defective myelination following Schwann cell specific Dicer depletion. Sciatic nerves of embryos (E17) and newborn mice (p4) were analyzed by electron microscopy for morphological effects of Schwann cell-specific Dicer depletion (**A**), and by quantitative RT-PCR for mRNA expression of factors known to regulate myelination. Number of biological replicates used: Dicer^fl/fl^ Dhh-Cre^+^ E17 n = 4, Dicer^wt/fl^ Dhh-Cre^+^ E17 n = 3, Dicer^fl/fl^ Dhh-Cre^+^ p4 n = 4, and Dicer^wt/fl^ Dhh-Cre^+^ p4 n = 4 (**B–D**). No morphological difference was observed before the onset of myelination at E17 between Dicer^wt/fl^ Dhh-Cre^+^ and Dicer^fl/fl^ Dhh-Cre^+^ nerves. By p4, myelination had begun in Dicer^wt/fl^ Dhh-Cre^+^ mice, but not in Dicer^fl/fl^ Dhh-Cre^+^ nerves. Some unsorted fibers were visible in Dicer^fl/fl^ Dhh-Cre^+^ nerves. Scale bars = 2 µm (**A**). Quantitative RT-PCR for mRNA expression in Dicer mutant nerves showed lack of developmental upregulation for activators of myelination. P values for comparisons between p4 controls and mutants were: *p* = 0.0026 (Oct6), *p* = 0.0028 (Egr2), *p* = 0.0003 (Brn2), *p* = 0.0028 (Sox10; **B**), no significant difference in expression of suppressors of myelination Sox2 and c-jun (**C**) and altered expression of Notch signaling components and p75NTR. P values for comparisons between p4 controls and mutants were: *p* = 0.061 (Delta1), *p* = 0.043 (Jagged1), *p* = 0.0001 (Jagged2), *p* = 0.02 (Notch3), *p* = 0.0061 (p75NTR; **D**). Dicer mRNA expression was significantly upregulated upon myelination in Dicer^wt/fl^ Dhh-Cre^+^ sciatic nerves (p4 in comparison with E17, *p* = 0.0004). At both time points, E17 and p4, significant Dicer mRNA depletion in Dicer^fl/fl^ Dhh-Cre^+^ was shown (E17: *p* = 0.0007; p4: *p*<0.0001; **E**). Presence of the recombined Dicer allele in E17 and p4 mice was demonstrated by PCR using primers that differentiate between wild type Dicer and the recombined allele (**F**). The wild type allele is 1.3 kb in length, and the recombined allele is approx. 500bp (**G**). Akt phosphorylation and expression were analyzed by Western blot in Dicer^wt/fl^ Dhh-Cre^+^ compared to Dicer^fl/fl^ Dhh-Cre^+^ at p4. P-Akt in relation to total Akt was reduced to 62±14% of control level in Dicer^fl/fl^ Dhh-Cre^+^ at p4. All p values were determined using an unpaired two-tailed student's t-test. All error bars indicate standard deviation.

Next, we performed a time course analysis of changes in Dicer mutant nerves (**Fig. 3**). At four days of age (p4), EM analysis of sciatic nerves showed myelin formation in control Dicer^wt/fl^ Dhh-Cre^+^. In contrast, no myelinating Schwann cells were observed in Dicer mutant nerves of age-matched Dicer^fl/fl^ Dhh-Cre^+^ littermates. As in the 18-day old mice, proper radial sorting with 1∶1 Schwann cell-to-axon ratio was observed in most fibers at p4 (**Fig. 3a**). In contrast, EM analysis of sciatic nerves from Dicer^fl/fl^ Dhh-Cre^+^ versus Dicer^wt/fl^ Dhh-Cre^+^ mice at the age of 17 embryonic days (E17) revealed no structural difference (**Fig. 3a**). Dicer expression is upregulated upon myelination in control p4 nerves compared to control E17 nerves. In Dicer^fl/fl^ Dhh-Cre^+^ at E17 and at p4, Dicer depletion was found by quantitative RT-PCR (**Fig. 3e**). Furthermore, presence of the inactivated Dicer^flox^ allele was shown by PCR (**Fig. 3f**).

### The expression of specific miRNAs is altered in Dicer-deficient peripheral nerves

To identify specific miRNAs involved in peripheral nerve myelination, we performed a differential microarray analysis of miRNA extracted from Dicer^fl/fl^ Dhh-Cre^+^ and Dicer^wt/fl^ Dhh-Cre^+^ nerves. Since the Dhh promoter is specifically active in Schwann cells, we would expect Dicer to be ablated exclusively in this cell type (not neurons or other cell types in the analyzed nerve) and the microarray to identify miRNAs specifically expressed in Schwann cells. We chose two different developmental time points for analysis. The first time point was before the onset of myelination and before structural differences could be observed between Dicer^fl/fl^ Dhh-Cre^+^ and Dicer^wt/fl^ Dhh-Cre^+^ nerves (at E17). The second time point was p4, when myelination had already started in control mice and was already evidently impaired in Dicer^fl/fl^ Dhh-Cre^+^ mice.

**Figure 4:**
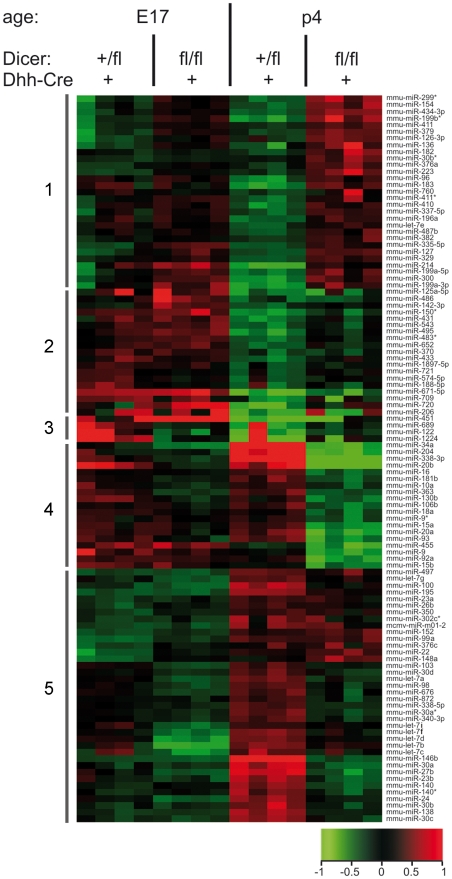
Heat map of developmentally and/or Dicer dependently regulated miRNAs in sciatic nerves. Expression of miRNAs in sciatic nerves of E17 and p4 Dicer^fl/fl^ Dhh-Cre^+^ and control Dicer^wt/fl^ Dhh-Cre^+^ littermates was analyzed by miRNA microarray. Four biological replicates for each group were analyzed on separate arrays. Of the 216 miRNAs expressed, 109 miRNAs (listed on the right side of the heat map) were differentially expressed, either in an age-dependent manner or in a manner dependent on the expression of Dicer in Schwann cells (*p*≤0.05, log_2_ ratio≥0.5). Differential expression in log_2_ ratio is color coded as indicated in the legend below the heat map (red = upregulation, green = downregulation). Based on hierarchical clustering, five groups, each containing miRNAs of similar expression pattern are indicated by gray bars on the left side. Group 1 contains miRNAs which are upregulated in Dicer^fl/fl^ Dhh-Cre^+^ compared to Dicer^wt/fl^ Dhh-Cre^+^ nerves at E17 and at p4. Group 2 contains miRNAs that are downregulated in both genotypes at p4 compared to E17. Group 3 includes miRNAs that are downregulated in Dicer^wt/fl^ Dhh-Cre^+^ nerves at p4 when compared to E17 and downregualted in Dicer^fl/fl^ Dhh-Cre^+^ compared to Dicer^wt/fl^ Dhh-Cre^+^ nerves at E17. Group 4 contains miRNAs which are downregulated in Dicer^fl/fl^ Dhh-Cre^+^ compared to Dicer^wt/fl^ Dhh-Cre^+^ nerves at p4 and are abundantly expressed also in Dicer^wt/fl^ Dhh-Cre^+^ nerves at E17. Group 5 includes miRNAs that are upregulated in Dicer^wt/fl^ Dhh-Cre^+^ nerves at p4 compared to both, Dicer^fl/fl^ Dhh-Cre^+^ and Dicer^wt/fl^ Dhh-Cre^+^ nerves at E17.

**Figure 5:**
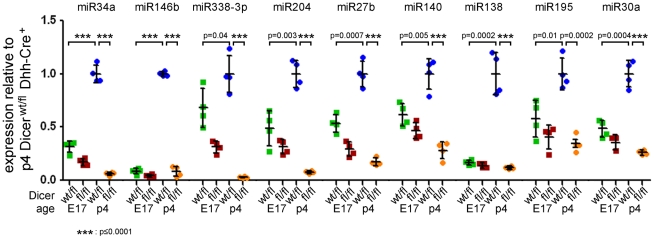
Real time PCR confirmation of miRNAs upregulated upon myelination. Expression of miRNAs in sciatic nerves of E17 and p4 Dicer^fl/fl^ Dhh-Cre^+^ (fl/fl) and control Dicer^wt/fl^ Dhh-Cre^+^ (wt/fl) mice was analyzed by real time PCR with Taqman probes. The levels of miRNA expression were quantified in comparison with sno234 RNA as the endogenous control. Expression is shown as relative values compared to p4 Dicer^wt/fl^ Dhh-Cre^+^. Error bars indicate standard deviation. All miRNAs analyzed were significantly downregulated in Dicer^fl/fl^ Dhh-Cre^+^ nerves at p4: *p*≤0.0001 (miR-34a, miR-146b, miR-338-3p, miR-204, miR-27b, miR-140, miR-138, miR-30a), *p* = 0.0002 (miR-195). Furthermore, miRNAs were confirmed to be upregulated upon myelination: *p*≤0.0001 (miR-34a, miR-146b), *p* = 0.04 (miR-338-3p), *p* = 0.003 (miR-204), *p* = 0.0007 (miR-27b), *p* = 0.005 (miR-140), *p* = 0.0002 (miR-138), *p* = 0.01 (miR-195), *p* = 0.0004 (miR-30a). P values were determined using unpaired two-tailed student's t-test.

Among the 216 miRNAs expressed in peripheral nerves, we identified a total of 109 miRNAs which were either significantly developmentally up- or downregulated (p4 compared to E17) or significantly different between controls and mutants (**Fig. 4**). For the observed phenotype, however, the miRNAs of major interest were those which displayed an upregulation upon the onset of myelination and were also significantly downregulated in p4 Dicer mutant nerves compared to controls. The sixteen miRNAs which fulfilled these two criteria mentioned above are listed (**Table 1**). For nine of these miRNA we could confirm the differential expression by real time PCR using miRNA specific Taqman probes (**Fig. 5**). Since downregulation of Schwann cell miRNAs might also control important steps of proper myelination, we analyzed the microarray dataset for those miRNAs that were significantly downregulated upon myelination and significantly reduced by ablation of Dicer from Schwann cells (either at E17 or at p4). Only three microRNAs met these criteria: miR-9, miR-455, and miR-1224.

**Table 1:** miRNAs significantly upregulated in sciatic nerves during myelination log_2_ ratio ≥0.5 and significantly decreased as a consequence of Schwann cell specific Dicer depletion log_2_ ratio ≤−0.5.

Agilent Systematicname	effect of Schwann cell specificDicer depletion in P4 [pValue]	effect of Schwann cell specificDicer depletion in P4 [log2 Ratio]	myelination effect inDicer +/− Dhh-Cre+ [pValue]	myelination effect inDicer +/− Dhh-Cre+ [log2 Ratio]
mmu-miR-338-3p	0.0000024	−3.01	0.00087	0.96
mmu-miR-338-3p	0.000010	−2.15	0.00067	1.05
mmu-miR-34a	0.00000046	−2.31	0.0000039	1.55
mmu-miR-34a	0.00000041	−2.20	0.0000061	1.52
mmu-miR-204	0.000010	−2.28	0.0025	1.11
mmu-miR-204	0.000010	−1.55	0.0011	0.92
mmu-miR-146b	0.00000031	−2.25	0.00000023	2.14
mmu-miR-146b	0.000020	−0.69	0.0000071	0.70
mmu-miR-27b	0.0000040	−1.55	0.00019	0.97
mmu-miR-27b	0.000074	−1.00	0.00025	0.71
mmu-miR-30a	0.000024	−1.35	0.00015	1.05
mmu-miR-30a	0.000026	−1.23	0.00016	0.98
mmu-miR-30a	0.000090	−0.98	0.023	0.64
mmu-miR-140	0.000035	−1.07	0.00015	0.83
mmu-miR-140	0.0000035	−0.81	0.0000089	0.65
mmu-miR-23b	0.000092	−1.00	0.0019	0.55
mmu-miR-23b	0.00018	−0.98	0.0036	0.80
mmu-miR-138	0.0000038	−0.90	0.000017	1.01
mmu-miR-138	0.000069	−0.69	0.000091	0.74
mmu-miR-30a*	0.000027	−0.88	0.00021	0.61
mmu-miR-100	0.00048	−0.84	0.000047	0.92
mmu-miR-100	0.00039	−0.57	0.000075	0.95
mmu-miR-140*	0.00089	−0.78	0.00073	0.69
mmu-miR-30c	0.00044	−0.77	0.000044	0.79
mmu-miR-30b	0.00080	−0.77	0.00088	0.75
mmu-miR-24	0.00017	−0.72	0.00039	0.54
mmu-miR-24	0.000048	−0.67	0.0014	0.67
mmu-miR-195	0.0000083	−0.57	0.00000067	1.31

### Altered myelination signals in Dicer-deficient peripheral nerves

In order to determine the effect of miRNA depletion from Schwann cells on the expression of molecules involved in peripheral nerve myelination, we performed quantitative RT-PCR on mRNA isolated from E17 and p4 Dicer^fl/fl^ Dhh-Cre^+^ and Dicer^wt/fl^ Dhh-Cre^+^ nerves. The mRNA expression of several transcription factors, cell surface receptors, and other molecules known to be involved in peripheral nerve myelination was significantly altered in p4 Dicer^fl/fl^ Dhh-Cre^+^ nerves compared to Dicer^wt/fl^ Dhh-Cre^+^ (**Fig 3b–d**). The factors known to promote myelination, Oct6, Egr2, Brn2, and Sox10, were all significantly downregulated in Dicer mutant nerves. Apart from Brn2, dicerless nerves from 4-day old mice expressed these factors at levels similar to embryonic nerves from E17 mice (**Fig. 3b**). Inhibitors of myelination, including Sox2 and c-Jun were not altered (**Fig. 3c**). We also observed dysregulation of components of the Notch signaling pathway, including significant downregulation of Notch3 and Jagged2, as well as a somewhat lower Delta1 expression (which did not attain statistical significance) in Dicer mutant nerves. In contrast, Jagged1 was upregulated in Dicer mutant nerves. Therefore, loss of miRNA expression in peripheral nerves dramatically alters the balance between pro- and anti-myelin signals.

## Discussion

**Figure 6:**
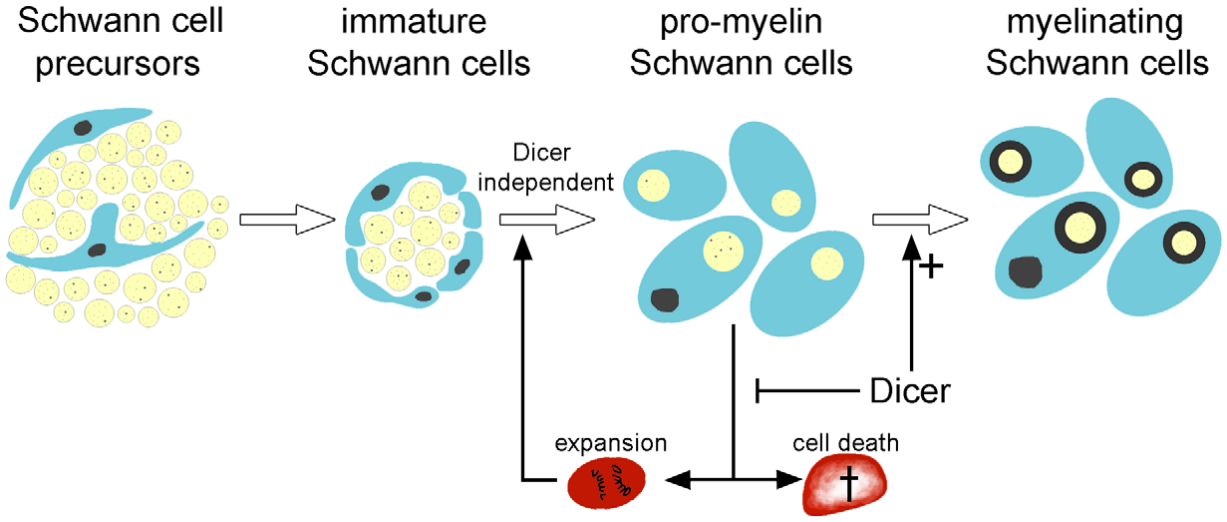
The role of Dicer and miRNAs in Schwann cell development. In the absence of Dicer, Schwann cells develop normally to the pro-myelin stage with properly sorted axons (light yellow). The presence of Dicer is required for acquisition of myelination competence by Schwann cells (turquoise). When Dicer is removed from Schwann cells (red), reduction of miRNAs leads to Schwann cell hyperproliferation and degeneration, suggesting that miRNAs are essential both for driving maturing Schwann cells into cell cycle arrest and terminal differentiation, as well as for their survival.

Here we show that Dicer expression in developing Schwann cells is crucially involved in peripheral myelination. By using a different, independently generated conditional Dicer knockout mouse strain [17], [18], we confirm recently published data [14]. The morphological and ultrastructural changes in dicerless peripheral nerves points to a crucial role for miRNAs in the transition of Schwann cells from the pro-myelin stage to the myelinating stage. Although Dicer is depleted at earlier developmental stages in Schwann cell precursors of Dicer^fl/fl^ Dhh-Cre^+^ mice, Dicer-deficient Schwann cells are nevertheless able to reach the immature Schwann cell stage, as evidenced by positive S100 expression and basal lamina formation in Dicer^fl/fl^ Dhh-Cre^+^ nerves and despite an altered microRNA expression profile already evident at E17. Proper sorting of the majority of axons indicate that Schwann cell differentiation into the myelinating phenotype is mainly arrested at the pro-myelin stage at the time when Dicer expression begins to increase in control sciatic nerves (**Fig. 6**). The reduction in mature myelin by ultrastructural or biochemical analysis (CNPase, MBP, PMP22) supports this conclusion. Some fibers appeared to overcome the myelination block, possibly due to incomplete recombination or due to the presence of residual Dicer protein that persists after genetic ablation. However, the myelin sheaths formed around these nerves are abnormally thin, confirming previous findings [14]. Dicer-deficient Schwann cells not only failed to myelinate, but also were unable to form normal Remak bundles of unmyelinated small-caliber axons. Cre expression itself has been shown to be toxic to certain cell types [19]. To exclude that toxicity of Cre expression in Schwann cells induced myelination defects or miRNA expression changes, we used Dicer^wt/fl^ Dhh-Cre^+^ littermates as controls. We did not see any evidence for spurious effects on myelination due to Cre expression.

Myelin formation is known to be associated with cessation of Schwann cell proliferation. In 22-day old Dicer^fl/fl^ Dhh-Cre^+^ nerves, we observed mitotic events within Schwann cell nuclei and an increased proliferation rate as determined by Ki67 staining. The increased cell proliferation observed in arrested immature Schwann cells was not seen by others, as determined by BrdU incorporation rates in younger mice only [14]. This difference may reflect the age difference of the analyzed mice. In contrast to the result of the previous study, our data suggests that increased Schwann cell proliferation is indeed a consequence of Dicer ablation from Schwann cells and accompanies the defect in myelination, at least at an older age. Consistently with Pereira et al., we also observed a slight increase in the number of TUNEL-positive cells in sciatic nerves of Dicer mutant mice.

How can the above findings be mechanistically explained? In Dicer-less Schwann cells, a global reduction of all miRNAs may directly lead to both Schwann cell proliferation and death, maybe because certain miRNAs are necessary for exiting the cell cycle, terminal differentiation, and cell survival (**Fig. 6**). It is not necessarily contradictory that both cell death and cell proliferation are stimulated in the absence of Dicer, since its global effect on all miRNAs is expected to produce pleiotropic phenotypes. Alternatively, in the absence of Dicer, arrested pro-myelin Schwann cells may become prone to degeneration. Cell death might induce compensatory proliferation, either of Schwann cells lacking Dicer or of Schwann cells in which recombination of the Dicer^flox^ allele has failed. However, it is not obvious which signalling pathway might trigger such a hypothetical compensation. Also, the lower percentage of dying cells (2%) compared to the higher percentage of proliferating cells (11%) argues against compensatory proliferation.

By microRNA microarray, we identified numerous miRNAs that are expressed in peripheral nerves during development at E17 and p4. Among the 216 expressed miRNA, 109 were either up- or downregulated upon differentiation of immature Schwann cells at E17 into myelinating Schwann cells at p4, or differentially expressed as a consequence of Dicer depletion at E17 or at p4. Unexpectedly, for a number of miRNAs, Dicer mutant Dicer^fl/fl^ Dhh-Cre^+^ nerves showed a higher expression when compared to control Dicer^wt/fl^ Dhh-Cre^+^ nerves. This suggests that other endoneural cells like fibroblast and endothelial cells or axonally transported miRNAs may contribute to the overall pool of miRNAs which are regulated in response to Schwann cell-specific lack of miRNA expression.

It is plausible to assume that miRNAs crucially involved in myelination should be upregulated as Schwann cell development progresses towards the myelinating phenotype. We therefore selected those miRNAs that were both (1) significantly upregulated upon myelination and (2) significantly decreased upon Dicer depletion in Schwann cells. Only 16 miRNAs met these criteria (**Table 1**). Furthermore, we identified miR-9, miR-455, and miR-1224 as microRNAs downregulated upon myelination and reduced following Dicer ablation from Schwann cells. MiR-9 has previously been shown to regulate PMP22 expression in oligodendrocytes [13]. Assuming that PMP22 is also regulated by miR-9 in Schwann cells, the downregulation of this negative regulator of the major myelin protein PMP22 upon myelination should promote myelin formation.

Although downregulation of miRNAs might also be crucial during the development of peripheral myelin, such miRNAs are unlikely to be responsible for the observed phenotype following Dicer depletion in our study.

Some of the upregulated miRNAs are important for oligodendrocyte differentiation, e.g. miR-338 [11], [12] and miR-138 [11]. In addition, developmental upregulation of some miRNAs identified in our screen was observed in differentiating oligodendrocytes *in vitro*, including miR-146, miR-23b, miR-24, and miR-27b in one study [13] and miR-204, miR-27b and miR-100 very recently in another study [20]. However, some miRNAs identified have not been previously reported to be involved in myelination, including miR-195, miR-140, miR-34a, miR-30a, miR-30b, miR-30c, and miR-140*. Interestingly, miR-219, despite being independently identified by two different groups as a key regulator of oligodendrocyte differentiation and CNS myelination [11], [12], was not identified as being differentially expressed in Dicer^fl/fl^ Dhh-Cre^+^ peripheral nerves in our miRNA screen.

Another study analyzed and compared the miRNA expression pattern in proliferating and differentiated rat Schwann cells *in vitro* [21]. This study focused on downregulation of rno-miRNA-29a expression in differentiated Schwann cells and the negative regulatory effect of miRNA-29a on PMP22 expression. In our study, miRNA-29a was slightly but not significantly upregulated upon myelination, indicating that its inhibitory effect on PMP22 expression plays no major role in mice *in vivo* at this time point of development. The miRNAs identified by Verrier et al. to be upregulated in differentiated Schwann cells showed no overlap with our main candidates *in vivo* [21]. This is most likely caused by the differences in the experimental design. Under specific conditions, e.g. in nerve regeneration or at later time points of development, other miRNAs might be involved.

The process of myelination requires the specific upregulation of pro-myelinating proteins and the coordinated downregulation of anti-myelinating proteins at specific stages of myelin maturation. Interestingly, we found that several transcripts (Oct6, Egr2, Brn2, Sox10) encoding pro-myelinating proteins fail to be upregulated in Dicer^fl/fl^ Dhh-Cre^+^ nerves, with expression levels of Oct6, Egr2, and Sox10 in Dicer^fl/fl^ Dhh-Cre^+^ p4 nerves closely matching those in Dicer^wt/fl^ Dhh-Cre^+^ E17 nerves. The only exception was Brn2, which was further downregulated in Dicer^fl/fl^ Dhh-Cre^+^ p4 nerves than in E17 nerves. This is in contrast to the recent study by Pereira et al., where Sox10 expression was not significantly reduced and Oct6 expression was only marginally reduced. The dramatic downregulation of Egr2 reported by Pereira et al., on the other hand, was consistent with our study [14]. In addition, we found elevated levels of p75NTR mRNA in Dicer^fl/fl^ Dhh-Cre^+^ nerves, which is normally down-regulated after onset of myelination.

miRNA expression is normally associated with silencing of target mRNAs, either through enhanced mRNA degradation or translational inhibition of target transcripts. Therefore the Schwann cell differentiation defect observed in nerves of Dicer^fl/fl^ Dhh-Cre^+^ mice might reflect the failure of Dicer^fl/fl^ Dhh-Cre^+^ Schwann cells to downregulate anti-myelin signaling molecules. We tested the mRNA expression of the anti-myelinating factors Sox2 and c-Jun in Dicer^fl/fl^ Dhh-Cre^+^ versus Dicer^wt/fl^ Dhh-Cre^+^ nerves. We did not detect any significant difference in Sox2 or c-Jun mRNA expression between these two groups at the mRNA level; however, Pereira et al. reported elevated Sox2 in Dicer^fl/fl^ Dhh-Cre^+^ nerves [14].

miRNA-34a was recently shown to act as a tumor suppressor in human glioma cells by inhibiting cell proliferation [22], [23]. It has recently been shown that miRNA-34a is also downregulated in tumors of peripheral nerves called malignant peripheral nerve sheath tumors (MPNST) where it may act as a tumor suppressor as well [24]. MiRNA-34a was a main candidate in our screen and a major histological observation in Dicer mutant nerves was an increased Schwann cell proliferation. It is likely that miRNA-34a drives Schwann cell differentiation by shutting down their proliferation during development; failure of this regulatory circuit may be involved in the histogenesis of schwannomas. Besides miR-34a, other miRNAs that were identified in our screen were previously found to be associated with an inhibitory effect on proliferation of non-neural tumor cells, including miR-24 in HeLa cells [25] and miR-100 in oral squamous cell carcinoma [26].

Furthermore, we observed an increased phosphorylation of Erk in Dicer mutant nerves at p20. Activation of the Erk pathway is known to induce dedifferentiation of Schwann cells and Schwann cell proliferation, and might contribute to the observed failure of Schwann cells to myelinate [27], [28]. Also, overexpression of Ras protein can induce Schwann cell differentiation and proliferation arrest, in contrast to its proliferation-promoting effects on other cell types [29], [30]. In Dicer mutant nerves, we observed a significantly lower Ras expression compared to control nerves. Therefore, low levels of Ras might also contribute to the observed phenotype.

We also found altered mRNA expression of transcripts involved in Notch signaling, including Jagged1, Jagged2, and Notch3. In addition, Pereira et al. observed an increased expression of Notch1, and Hes1, as well as a reduced level of ErbB2 in Dicer^fl/fl^ Dhh-Cre^+^ nerves [14]. Of note, Jagged1 and Notch1 which were upregulated at the mRNA level in Dicer^fl/fl^ Dhh-Cre^+^ nerves compared to Dicer^fl/fl^ Dhh-Cre^+^, were previously identified as a target of miR-34a, which we identified in our miRNA screen [22], [31]. Deregulated Notch and/or neuregulin signaling may therefore partly explain the failure of immature Schwann cells to upregulate some pro-myelin transcripts, such as Egr2. Consistently with Pereira et al. we observed impaired Akt phosphorylation at Ser-473 in Dicer mutant nerves at the onset of myelination (at p4/p5) [14]. In contrast, at p18 we observed increased Akt phosphorylation in Dicer mutant nerves. Hence in young mice deletion of Dicer led to a reduction of the pro-myelinating phosphorylation of Akt. Since Akt activation promotes myelination [32], increased Akt phosphorylation may reflect a compensatory upregulation of pro-myelinating signals in response to abnormally high levels of anti-myelinating factors in the absence of miRNA regulation at an older age. In any case, it seems as if the compensatory response in Dicer^fl/fl^ Dhh-Cre^+^ nerves is unable to override anti-myelination signals, as the nerves nevertheless fail to myelinate.

It will be interesting to determine in the future whether miRNAs that are upregulated in both CNS and PNS myelination play analogous roles and/or target the same proteins in these cell types. Conversely, miRNAs that are distinctly upregulated in either oligodendrocytes or Schwann cells may target proteins and/or control processes that are specific to either PNS or CNS myelination.

Clearly, miRNAs play a crucial role in the myelination process both in the CNS and the PNS. An important task that remains for future studies will be to positively identify Schwann cell-specific target transcripts of and the mode of regulation by miRNAs that are specifically upregulated in peripheral nerves during PNS myelination.

## Materials and Methods

### Mice and ethical statement

We housed mice and performed animal experiments in accordance with the Swiss Animal Protection Law and in compliance with the animal welfare regulations of the Canton of Zurich. The Committee on Animal Experimentation of the Cantonal Veterinary Office of the Canton of Zurich has specifically approved this study under license number 200/2007. Dicer^fl/fl^ mice were obtained from Jackson Laboratory (strain name: Dicer1^tm1Bdh^/J; stock number: 006001) [17]. Dhh-Cre mice were kindly provided by Dr. Dies Meijer [33]. Dicer^fl/fl^ mice were crossed to Dhh-Cre, and subsequently, F1 mice were bred again to Dicer^fl/fl^ mice to obtain Dicer^fl/fl^ Dhh-Cre^+^ mice. For identifying Dhh-Cre transgene positive mice, the following primers were used: *Cre* fw: ACC CTG TTA CGT ATA GCC GA, *Cre* rev: CTC CGG TAT TGA AAC TCC AG. For distinguishing Dicer floxed and Dicer wild-type alleles, the following primers were used: DF1: CCT GAC AGT GAC GGT CCA AAG and DR1: CAT GAC TCT TCA ACT CAA ACT, producing a wild-type allele-specific product of 350bp and a floxed allele-specific product of 420bp. The recombined allele was amplified using DF1 primer and Ddel primer: CCT GAG CAA GGC AAG TCA TTC, the same primer set recognized also the wild-type allele (product size 1.3 kb).

### Electron microscopy

Mice at the age of p4 or older were anesthetized and transcardially perfused with PBS followed by 3.9% glutaraldehyde in 0.1 M phosphate buffer, pH 7.4. Sciatic nerves of embryos (E17) were fixed with glutaraldehyde in situ for at least 5 minutes. At least n = 4 mice of each genotype and age group (E17, p4, and p18, mixed gender) were analyzed. All nerves were then postfixed in glutaraldehyde in a test tube. Tissues were embedded in Epon using standard procedures. Semithin sections were stained with toluidine blue. Ultrathin sections were mounted on copper grids coated with Formvar membrane and contrasted with uranyl acetate/lead citrate. We examined the specimens using a Hitachi H-7650 transmission electron microscope operating at 80 kV. We took pictures with a digital CCD camera.

### miRNA microarray

miRNA was extracted from sciatic nerves as described in the next section. Sciatic nerves of embryos at the gestational age of 17 days (E17) or newborns at the age of 4 days (p4) were used. For each time point, 4 pairs of Dicer^fl/fl^ Dhh-Cre^+^ littermates and Dicer^wt/fl^ Dhh-Cre^+^ were used and analyzed separately on individual arrays (Dicer^fl/fl^ Dhh-Cre^+^ E17 n = 4: 1 male, 3 females, Dicer^+/fl^ Dhh-Cre^+^ E17 n = 4: 1 male, 3 females, Dicer^fl/fl^ Dhh-Cre^+^ p4 n = 4: 1 male, 3 females, and Dicer^+/fl^ Dhh-Cre^+^ p4 n = 4: 2 males, 2 females). Purity and quality of the isolated total RNA was determined using a NanoDrop ND 1000 (NanoDrop Technologies) and a Bioanalyzer 2100 (Agilent) respectively. Only those samples with a 260 nm/280 nm ratio between 1.6–2.1 and a 28S/18S ratio within 1.5–2 were further processed. Fluorescent miRNA with a sample input of 100ng of total RNA was generated. This method involves the ligation of one Cyanine 3-pCp molecule to the 3′ end of an RNA molecule using a miRNA Complete Labeling and Hyb Kit (Agilent). The quality of Cy3- RNA was determined using a NanoDrop ND 1000. Only RNA samples with a dye incorporation rate >2 pmol/µg were considered for hybridization. Cy3-labeled RNA samples were mixed with an Agilent Blocking Solution and resuspended in Hybridization Buffer using a miRNA Complete Labeling and Hyb Kit (Agilent). Target RNA Samples (45µl) were hybridized to Mouse miRNA 8x15k OligoMicroarrays (Agilent P/N G4472A, Design ID 019119y) for 20h at 55°C. Arrays were then washed using Agilent GE Wash Buffers 1 and 2 (Agilent), according to the manufacturer's instructions. An Agilent Microarray Scanner (Agilent p/n G2565BA) was used to measure the fluorescent intensity emitted by the labeled target. Raw data processing was performed using the Agilent Scan Control and the Agilent Feature Extraction Software Version 10.5.1.1. Quality control measures were considered before performing the statistical analysis. These included inspection of the array hybridization pattern (absence of scratches, bubbles, areas of non hybridization), proper grid alignment and number of green feature non-uniformity outliers (below 100 for all samples). Expression data was analyzed using R/Bioconductor. Briefly, median spot signals were log2-transformed and normalized using quantile normalization. Differential expression was assessed using t-test and fold-change analysis. miRNAs flagged as absent by the Feature Extraction Software in more than 50% of the samples in each of the 4 conditions were excluded from the results. All data is MIAME compliant and the raw data is available in the GEO archive under the accession GSE22023. For hierarchical clustering of the heatmap, we used as distance measure the euklidean distance of the normalized expression profiles of the miRNAs. Clusters were linked using the Ward's linkage rule that minimizes intra-cluster variance. A simplified version of the clustering tree is visualized on the left side of the heat map, including five groups of miRNAs.

### RNA extraction and real time PCR

RNA was extracted from sciatic nerves using miRNeasy (Qiagen) as described by the manufacturer using a polytron PT 3100 (kinematica). CDNA from mRNA was synthesized with QuantiTect, Reverse Transcription kit (Qiagen) and analyzed by real-time PCR using QuantiFast SYBR Green PCR kit (Qiagen). CDNA from miRNA was synthesized with TaqMan MicroRNA RT kit (Applied Biosystems) and analyzed using TaqMan MicroRNA Assays (Applied Biosystems) and Taqman Universal Master Mix II (Applied Biosystems). All samples were analyzed using a 7900HT Fast Real-Time PCR system (Applied Biosystems). Three to four biological replicates were used for each group analyzed (Dicer^fl/fl^ Dhh-Cre^+^ E17 n = 4: 3 males, 1 female, Dicer^+/fl^ Dhh-Cre^+^ E17 n = 3: 3 males, Dicer^fl/fl^ Dhh-Cre^+^ p4 n = 4: 3 males, 1 female, and Dicer^+/fl^ Dhh-Cre^+^ p4 n = 4: 2 males, 2 females). Of each biological replicate, two (mRNA) or three (miRNA) technical replicates were performed. The following primers were used: *c-jun* fw: CCT TCT ACG ACG ATG CCC TC, *c-jun* rev: GGT TCA AGG TCA TGC TCT GTT T; *Notch3* fw: CCA TCC TTG GAC TCA GGC, *Notch3* rev: AGC TGG TGT TAG TAG CTC C; *Jagged1* fw: GTT CTC CAA ATA ACT GTT CCC, *Jagged1* rev: ATT TCA TTC TGA CAG TGA CCC; *Jagged2* fw: TGC TGT CTG GCT TTG AAT GCC, *Jagged2* rev: AGC ATT AAG GCA CGG TTT CCC; *Delta1* fw: TTG TTC TTT CTC AGT GCC TCG, *Delta1* rev: CCC TTC TTG TTG ACG AAC TCC; *Sox2* fw: GCG GAG TGG AAA CTT TTG TCC, *Sox2* rev: CGGGAAGCGTGTACTTATCCTT; *Oct6* fw: TCG AGG TGG GTG TCA AAG G, *Oct6* rev: GGC GCA TAA ACG TCG TCC A, Egr2 fw: AAT GGC TTG GGA CTG ACT TG, Egr2 rev: GCC AGA GAA ACC TCC ATT CA; *Sox10* fw: AGA TGG GAA CCC AGA GCA C, *Sox10* rev: CTC TGT CTT TGG GGT GGT TG; *p75NTR* fw: CTA GGG GTG TCC TTT GGA GGT, *p75NTR* rev: CAG GGT TCA CAC ACG GTC T; *Brn2* fw: GCA GCG TCT AAC CAC TAC AGC, *Brn2* rev: GCG GTG ATC CAC TGG TGA G; *GAPDH* fw: CCA CCC CAG CAA GGA GAC T; *GAPDH* rev: GAA ATT GTG AGG GAG ATG CT; *Dicer* fw: ACC AGC GCT TAG AAT TCC TGG GAG; *Dicer* rev: GCA GCA GAC TTG GCG ATC CTG TAG.

### Western blot

Sciatic nerves were homogenized in nerve buffer (1% Triton×100, 137 mM NaCl, 2 mM EDTA, 20 mM Tris HCl, pH 8) using a polytron PT 3100 (kinematica). For analysis of 18- and 4-day old mice three different lysates were used, each containing nerves pooled from three mice with the respective genotype of mixed gender. Protein concentration was determined using the BCA protein assay (Pierce). Proteins were boiled in LDS (Invitrogen) containing β-mercaptoethananol, and loaded onto a NuPAGE® Novex Bis-Tris Gel (Invitrogen). After electrophoretical separation, proteins were blotted onto nitrocellulose membranes (Schleicher & Schuell) using XCell II Blot Module (Invitrogen). Membranes were blocked using TBST containing Top-Block (Sigma), and decorated with antibodies against CNPase (Abcam), MBP (Serotec), PMP22, and Ras (Abcam), Erk, phospho-Erk, Akt, phospho-Akt (Cell Signaling Technology), followed by incubation with the secondary anti-mouse IgG_1_ and IgG2a (Zymed) or anti-rabbit, and anti-rat IgG (Calbiochem). We visualized proteins using SuperSignal West Pico Chemiluminescent Substrate System (Pierce) and Amersham Hyperfilm ECL films (GE Healthcare).

### Immunohistochemistry

Sciatic nerves were fixed in 4% formalin and embedded in paraffin. For each genotype, at least four nerves were analyzed (22-day old Dicer^fl/fl^ Dhh-Cre^+^ and control Dicer^wt/fl^ Dhh-Cre^+^ littermates). Longitudinal paraffin or frozen sections were incubated with the following antibodies: anti-S100 (Dako), anti-MIB1 (Dako), B220/CD45R for B-cells (Pharmingen), CD3 for T-cells (clone SP7, NeoMarkers), CD68 for macrophages (Serotec) or stained with haematoxylin-eosin. For detection of primary antibodies, a Ventana machine was used according to the manufacturer's protocol. Mounted slides were analyzed on an Axiophot microscope (Zeiss), equipped with a JVC digital camera (KY-F70; 3CCD). Rabbit immunoglobulin fraction (Dako) served as negative control for S100 staining (data not shown). For assessing proliferation, 940–1400 nuclei per mouse were counted and the “MIB1 index” was determined as the percentage of nuclei positive for MIB1 immunohistochemistry. CD3- and CD68-positive cells were quantified in relation to total number of nuclei.
